# Pain-Related Worrying and Goal Preferences Determine Walking Persistence in Women with Fibromyalgia

**DOI:** 10.3390/ijerph19031513

**Published:** 2022-01-28

**Authors:** María Ángeles Pastor-Mira, Sofía López-Roig, Eva Toribio, Fermín Martínez-Zaragoza, Ainara Nardi-Rodríguez, Cecilia Peñacoba

**Affiliations:** 1 Department of Behavioral Sciences and Health, University Miguel Hernández, 03540 San Juan de Alicante, Spain; mapastor@umh.es (M.Á.P.-M.); f.martinez@umh.es (F.M.-Z.); anardi@umh.es (A.N.-R.); 2 Fibromyalgia Unit, Hospital of San Vicente del Raspeig, 03690 Alicante, Spain; toribio_eva@gva.es; 3 Department of Psychology, Rey Juan Carlos University, 28922 Madrid, Spain; cecilia.penacoba@urjc.es

**Keywords:** fibromyalgia, women, pain catastrophizing, pain-related worrying, goal preferences, walking, physical activity

## Abstract

Physical activity and exercise are relevant behaviors for fibromyalgia health outcomes; however, patients have difficulties undertaking and maintaining an active lifestyle. With a cross-sectional design, this study explored the role of pain-related worrying and goal preferences in the walking persistence of women with fibromyalgia. The sample included 111 women who attended a tertiary health setting. We adapted the Six-Minute Walk Test where participants decided either to stop or continue walking in five voluntary 6 min bouts. Women who were categorized higher in pain-related worrying reported higher preference for pain avoidance goals (*t* = −2.44, *p* = 0.02) and performed worse in the walking task (LongRank = 4.21; *p* = 0.04). Pain avoidance goal preference increased the likelihood of stopping after the first (*OR* = 1.443), second (*OR* = 1.493), and third (*OR* = 1.540) 6 min walking bout, and the risk of ending the walking activity during the 30 min task (*HR* = 1.02, [1.0–1.03]). Influence of pain-related worrying on total walking distance was mediated by goal preferences (*ab* = −3.25). In interventions targeting adherence in physical activity and exercise, special attention is needed for women who are particularly worried about pain to help decrease their preference for short-term pain avoidance goals relative to long-term goals such as being active through walking.

## 1. Introduction

Fibromyalgia is characterized by chronic widespread musculoskeletal pain and fatigue, among other symptoms such as nonrestorative sleep, anxiety, depression, and attention and memory disturbances. The etiology of fibromyalgia remains unknown, and its diagnosis is clinical and not based on objective tests [1,2,3]. Patients with fibromyalgia, usually show high functional impact and negative consequences in their quality of life, along with a high sociosanitary burden [2]. Regarding this health problem, with multiple psychological and physical symptoms, the best treatment approach includes physical, pharmacological, and psychological strategies [3,4], with the objective of improving physical and psychosocial functioning. For these patients, being physically active and doing exercise is widely recommended due to the positive effects it has on their physical function and quality of life among other health outcomes [4,5,6,7,8,9,10,11,12]. In fact, exercise is the initial recommended treatment in a graded strategy of intervention [4]. Walking is an effective form of exercise; it is easy and accessible with low musculoskeletal impact and it has been associated with improvements in cardiovascular disease risk factors, increasing aerobic capacity, reducing systolic and diastolic blood pressure, waist circumference, weight, and a decrease in both body fat and body mass index [13,14]. In addition, walking has shown positive effects on pain and physical function and it is recommended for people with chronic musculoskeletal pain such as fibromyalgia [15]. However, women with fibromyalgia are less physically active than others [16]. A high proportion present a sedentary lifestyle [10,17], impaired subjective and objective physical function [18], and low adherence to unsupervised walking exercise [19]. Exercise health benefits are undermined when patients do not maintain their routines [20]; however, long-term exercise adherence continues to be a clinical challenge when taking into account previous research [21,22]. In fact, some authors have suggested walking as a form of exercise along with strategies aimed at maintaining patients’ participation [15].

Both undertaking and maintaining exercise in chronic pain involve a self-regulation drive where motivational perspectives could be relevant. In line with the fear-avoidance model [23], people with pain can anticipate negative consequences from physical activity or exercise, such as a worsening of the pain. Furthermore, the fear associated with these expected consequences lead to avoiding physical activity or exercise in order to try to prevent pain. In fibromyalgia, some authors have shown that fear of movement and avoidance behavior toward physical activity is highly prevalent [24]. The fear of movement and the subsequent avoidance behavior often happens as a result of catastrophizing worries about pain and its consequences. The fear-avoidance model has been enhanced by adopting a motivational perspective, which states that people with chronic pain are faced with multiple competing goals and they should prioritize between those related to resolving and avoiding pain and those related to maintaining or incorporating other important goals different from pain control [25,26,27,28]. Strong achievement goals may reduce avoidance behavior [25,29] or increase patients’ persistence in painful physical activities [30]. In this vein, the preference for competing goals such as being active and pain avoidance could play a main role in undertaking and maintaining physical activity and exercise in patients with fibromyalgia.

From this motivational approach, pain catastrophizing would reflect the concern about pain interference in goals other than pain control and it would not be grounded in psychopathology approaches [25]. In this regard, Crombez et al. [31] have shown that the content of pain catastrophizing self-reported measures is closer to a pain-related worrying construct than a catastrophizing construct. On the other hand, the term “pain catastrophizing” has been labeled as negative and stigmatizing [32]. Due to these concerns and the suggestion of several authors [25,31] we will use the term “pain-related worrying” synonymously.

In women with fibromyalgia, pain is a reported obstacle for walking exercise [33] and the goal of avoiding it can compete with the goal of undertaking the exercise. This issue is more relevant for patients highly worried about their pain. Therefore, they are more likely to prefer pain avoidance goals than low pain catastrophizing patients, who would be more likely to get involved in long-term achievement goals, such as maintaining walking in the context of doing exercise (more goal persistence and, therefore, more adherence). Hence, pain catastrophizing could have an influence on exercise behavior through the patients’ goal preferences. The relevance of catastrophizing in chronic pain and fibromyalgia has been highlighted [34,35,36,37,38,39,40]. Recently, some authors have shown pain catastrophizing as a moderator in fibromyalgia pain intensity and physical activity relationships [41]. However, to the best of our knowledge, little is known about its role in undertaking and, mainly, maintaining walking exercise, taking into account its possible influence on habitual patients’ goal preferences.

Owing to the relevance of physical activity and exercise in fibromyalgia health outcomes, patients’ difficulties in maintaining exercise, and the influence of catastrophizing and goal preferences on physical activity, this study aimed to explore their joint role in the walking persistence in women with fibromyalgia. We expected that pain-related worrying would lead to an increased pain avoidance goal preference relative to physical activity and exercise achievement goals, and to less persistence in a walking physical activity task.

## 2. Materials and Methods

### 2.1. Participants

A total of 111 women who attended the Fibromyalgia Unit (FU) of the Valencian Community (San Vicente del Raspeig Hospital) participated in this study. The mean age was 51.4 (*SD* = 9.7). Most were married (69.4%; *n* = 77), with primary (46.8%; *n* = 52) or secondary studies (38.7%; *n =* 43). At the time of the study, 19.8% of participants worked outside the home (*n* = 22), 26.1% were on sick leave (*n* = 29), 25.2% were unemployed (*n* = 28), 23% were housewives (*n* = 23), and 7.2% were retired (*n* = 8). The mean time from the first symptoms was 14.1 years (*SD* = 9.8) and from diagnosis was 6.2 years (*SD* = 6.2). The mean pain intensity perception was 6.8 (out of 10; *SD* = 1.7) and the mean of the perceived fibromyalgia impact was 72.3 (out of 100; *SD* = 15.8) (Table 1).

### 2.2. Variables and Instruments

Sociodemographic and clinical variables were measured with ad hoc questions.

Pain catastrophizing: We used the total score of the Spanish validation [40] of the Pain Catastrophizing Scale (PCS) [42]. This scale contains 13 items answered on a 5-point Likert scale from 0 (not at all) to 4 (all the time) (rank 0–52) and assesses three dimensions: rumination (focusing excessively on pain sensations), magnification (tendency to magnify the threat value of pain sensations), and helplessness (to perceive oneself as unable to control pain). A higher PCS total score represents higher pain catastrophizing (*α* = 0.95). As highlighted in the introduction, recent research has shown the content of this construct is better represented by pain-related worrying [31].

Pain intensity: Measured with the mean score of the maximum, minimum, and usual pain intensity during the last week and pain intensity at time of the assessment. Items were answered with an 11-point numerical rating scale (0 = “no pain at all” and 10 = “the worst pain you can imagine”) adapted from Jensen et al. [43]. The scale has shown strong psychometric properties in Spanish FM samples [44,45]. High mean scores indicate high pain intensity (*α* = 0.86). As the pain intensity at the time of the assessment may determine the walking performance more than the general pain intensity, we also used this single item as another measure of pain (pain intensity before the task).

Fatigue intensity before the task: With a single item answered on an 11-point numerical rating scale (0 = “no fatigue at all” and 10 = “the worst fatigue you can imagine”) we measured fatigue perception before starting the walking task.

Goal preferences: We used the adapted version for physical activity and exercise (GPQ-PA) [46] of the Spanish version [47] of the Goal Pursuit Questionnaire (GPQ) [29]. The GPQ assesses the habitual goal pursuit of people with pain, taking into account pain avoidance short-term goals relative to achievement or long-term goals (related to different tasks), which can be activated at the same time in one situation. Following the same GPQ design and wording [29], we considered physical activity and exercise as achievement goals in five physical activity situations. Therefore, the GPQ-PA is comprised of five items that each include a vignette with a situation related to physical activity and exercise: (1) walking while taking advantage of other daily activities, (2) brisk walking for exercising at least 30 min, and undertaking (3) light, (4) moderate, and (5) vigorous physical activity intensities with the aim of exercising. The participants must imagine themselves, as vividly as possible, in the presented situation. Each vignette (i.e.,: “*While you are walking taking advantage of going to work, shopping or taking the dog out, your body becomes increasingly painful. You are expected to complete your walking route today”)* is followed by a sentence showing a thought which indicates a goal preference (i.e.,: *“I think it is more important to reduce my pain now than to finish this walking route”*) that the participant must rate on a 6-point Likert scale (1 = strongly disagree, 6 = strongly agree). The more agreement, the higher the preference for avoiding pain (short-term goal) versus finishing the activity (long-term goal). Higher mean scores indicate stronger preferences for pain avoidance goals relative to physical activity and exercise achievement goals. The GPQ-PA internal consistency in this sample was *α* = 0.87.

Fibromyalgia impact: We used the Spanish adaptation of the Revised Fibromyalgia Impact Questionnaire (FIQ-R) [48] to measure the perceived health impact of fibromyalgia, in terms of function (9 items), overall impact (2 items), and symptoms (10 items). Items are answered on an 11-point numerical rating scale from 0 to 10, with different wording anchors depending on the item. The total score of fibromyalgia impact is also obtained (rank 0–100). Higher scores represent a higher impact on quality of life (*α* = 0.90).

Physical activity: Total MET minutes/week was used as an indicator of previous physical activity, measured by the International Physical Activity Questionnaire-Short Form (IPAQ-S) [49]. Items ask respondents to report the frequency and duration of walking, moderate and vigorous physical activity intensity, performed for at least 10 min per session in the last week. We used the total MET minutes/week, which is the score of metabolic equivalence of minutes/week spent overall on physical activity.

Walking persistence: We used the Six-Minute Walk Test (6MWT), which is a clinically relevant objective measure of the physical function recommended in fibromyalgia [50] and is regarded as a feasible and valid submaximal exercise test [51]. It records the distance that each participant can quickly walk on a 30 m flat surface in a period of 6 min. We adapted the 6MWT in order to get a measure of walking persistence taking into account the content of the task-contingent persistence behavioral pattern, which is defined as “behavioral persistence in finishing tasks or activities despite pain” and is considered to be an adaptive behavioral activity pattern different from the excessive persistence [52,53]. We adopt this walking task measure as a proxy of adherence.

In our adaptation (see design section), participants decide to stop or continue walking in each bout of 6 min with a maximum duration of 30 min (for five bouts). We measure three variables as different indicators of walking persistence: (1) decision making: stopping vs. following walking through each 6 min bouts was used to operationalize the conscious decision aimed at persistence in walking; (2) total number of complete laps (each lap 60 m), which allows us to conduct a survival analysis (see statistics section) but is more inaccurate than the (3) total walking distance, which is registered as a continuous variable (total meters walked, including those in the last possible incomplete lap). The two latter variables were used to operationalize performance in the walking task.

When participants finished the walking task, they were asked for their reasons for definitively leaving the test.

### 2.3. Design and Procedure

This research was approved by the Ethic Committees of the Alicante General Hospital and the Miguel Hernández University and corresponds to the third study of a broader work, which aims to identify a physical activity and exercise self-regulation model in women with fibromyalgia in rehabilitation settings. Informed consent was obtained from all subjects involved in the study. Inclusion criteria was: women, aged between 18 and 70 years, with a fibromyalgia diagnosis confirmed by the physicians of the FU, following ARA criteria [54], with the ability to properly fill out the self-reported measures, without problems or comorbidity preventing walking and with the adequate physical conditions to perform the 6MWT (contraindications include unstable angina or myocardial infarction in the previous month, resting heart rate of >120, systolic blood pressure of 180 mmHg, or diastolic blood pressure >100 mmHg, and oxygen saturation of >90) [55]. A consecutive selection of the 121 FU patients who met the inclusion criteria was performed; they were invited to participate in the study and 118 accepted and signed the informed consent. Of those women, 3 patients did not meet the specific criteria to perform the 6MWT, 2 patients refused to continue once the task started (one woman felt anxiety during the task, and one stopped because she had no more time), and 2 patients’ data were eliminated due to mistakes in the data recording. Therefore, the sample was composed of 111 women. The period of recruitment spanned from July 2019 to February 2020.

### 2.4. Research Design

This is an observational cross-sectional study performed at the FU setting. Research design was thought (1) to establish a valued goal context for patients, and (2) to resemble a test of walking persistence, based on individual decision.

Both at the first and the last individual medical interview in the FU, patients were examined by the doctor to test the standard criteria for the 6MWT performance. Likewise, the doctor informed about the 6MWT as another component of the patients’ assessment in the FU, provided motivational instructions about the relevance of the 6MWT task for their assessment and advised patients to walk as far as they could and in the longest time possible between 6 min and 30 min (valued goal context). The time of 30 min was established as it is the minimum walking exercise time recommended for women with fibromyalgia in our research [56].

Afterwards, the patients completed the questionnaires with the help of the researcher (pre-test assessment) and performed the 6MWT. Following the standard administration, they were instructed to walk as far as they could and as fast as they could, but in a way that was comfortable to them. Therefore, the women decided their walking speed and, as is usual in the 6MWT, took minor breaks during the task. We used the standard 6MWT application sequence. However, we slightly modified the instructions at the end of every 6 min walking bout, asking patients if they wanted to do another 6 min of walking (*“…could you repeat 6 min more, do you want to do it?*”) until a maximum of five bouts had been completed.

The researchers who applied the 6MWT were different from those who were applying the pre-test assessment. They were unaware of the research hypothesis. The researchers who applied the 6MWT were also unaware of the scores in pain catastrophizing, goal preferences, and the other measures obtained in the pre-test.

### 2.5. Statistics

The SPSS v25 was used for analysis. Firstly, we performed descriptive, parametric, and nonparametric analyses of differences between women who were categorized as high and low based on their scores in PCS. Although PCS is designed to assess pain catastrophizing as a continuous variable, some authors have used a median split approach to identify high and low pain-catastrophizing patients. Taking into account the score distribution in our sample and the data reported by other authors [38,57,58,59], we used the median-split criteria only for comparison analysis, where scores ≤31 and >31 indicate low and high pain catastrophizing or pain-related worrying, respectively.

Secondly, logistic regression analyses were conducted to explore the differences between patients who decided to stop vs. those who followed through walking at the end of each 6 min bout (decision making). Hence, the binary outcome variable was stopping vs. continuing walking. Odds ratios (*OR*) and 95% CI were calculated. In addition, we examined pain-related worrying (using PCS total score) and goal preferences controlling sociodemographic, physical activity, and clinical variables. For each bout, we carried out a preselection process of variables with univariate analyses exploring age, educational level, occupational status, civil status, duration of problem, time since diagnosis, overall pain intensity, pain and fatigue intensity before the task, fibromyalgia impact, and MET minutes/week. Variables associated with outcome at *p* (Wald) values <0.20 in univariate analyses were included in a multivariate logistic model. As a result of the high correlation between measures and the Tolerance and VIF index values, the collinearity effects were controlled, including fibromyalgia impact and removing pain intensity. We used the forward and backward step likelihood ratio (LR) method as it has been recommended to select the best model applying the criteria of Mallow’s Cp adapted by Hosmer and Lemeshow [60].

Thirdly, using the total number of laps variable, we performed a survival analysis to explore the association between catastrophizing and the probability of following through with walking along the 30 min of the task. Stopping walking was specified as the “event” and the total number of laps walked was specified as “survival time”. Women who reached the 30 min walking distance at the end of the test were considered “free from the event” and, then, as “right censored observations”. We carried out two survival analysis: (1) the Kaplan–Meier method to estimate the walking survival functions, comparing the equality of walking survival distributions for the high and low pain catastrophizing groups using the LongRank test (Mantel–Haenszel); and (2) a multivariate Cox regression analysis was performed to model the hazard function to predict the probability of stopping at a number of laps. We introduced pain-related worrying and goal preferences, controlling sociodemographic and clinical variables. These variables were selected taking into account that *p* ≤ 0.20 in previous univariate was applied. A forward stepwise was the method to estimate the final model and the p value of each variable with the likelihood ratio test.

Finally, with the walking distance variable, we determined to what extent pain-related worrying and goal preference influenced total walking distance covered, using the PROCESS macro, version 3.4.1 [61]. A simple mediation model (model 4) was tested with goal preference as a mediator, controlling sociodemographic, physical activity, and clinical variables. Inference about the direct effect of catastrophizing on total walking distance is framed in terms of a null hypothesis test.

Goal preference is formulated by constructing a bootstrap confidence interval, using the bootstrapping method with 10.000 samples and seeding the random number generator.

## 3. Results

Table 1 shows descriptive and difference analysis between patients with low and high scores on pain-related worrying. The mean score of pain-related worrying was 30.9. Significant differences between groups were found on pain intensity (*t* = −4.68, *p* = 0.0001), fibromyalgia impact (*t* = −4.46, *p* = 0.0001), goal preferences (*t* = −2.44, *p* = 0.02), total walking distance (*t* = 2.05, *p* = 0.04), and number of laps (*t* = 2.07, *p* = 0.04).

### 3.1. Decision Making about Stopping or Following through with Walking Each 6 Min Bout

All participants walked the first 6 min bout. Forty-six women (41.4%) decided to stop the task at the end of the first bout and 65 women followed through with the second bout. Only 13 women (11.7%) completed the 30 min task (Table 1). The three most frequent reasons for leaving the test were fatigue (40.0%), pain (25.5%), and poor physical conditions (dizziness, difficulties with breathing, and trembling, among others) (11.9%). Pain was a more common reason in the high pain pain-related worrying group (37.5%, *n* = 21) than in the low pain group (12.7%, *n* = 7).

Univariate logistic analysis results showed selected goal preferences, pain-related worrying, educational level, fibromyalgia impact, pain and fatigue before task, and MET minutes/week. Results of the step methods and the selection of the best predictive model showed that the more pain-avoidance goal preferences (*OR* = 1.443 [1.026–2.030]), the more fibromyalgia impact (*OR*= 1.029 [1.000–1.060]) and a higher educational level (*OR* = 2.428 [1.055–5.589]) increased the probability of stopping vs. walking after the first bout.

Goal preferences was the only variable which increased the likelihood of stopping after the second (*OR* = 1.493 [1.092–2.042]) and the third bout (*OR* = 1.540 [1.073–2.210]). No significant predictors were found after the fourth bout (Table 2).

### 3.2. Walking Performance along the Walking Task

#### 3.2.1. Total Number of Complete Laps: Survival Analysis

**Figure 1:**
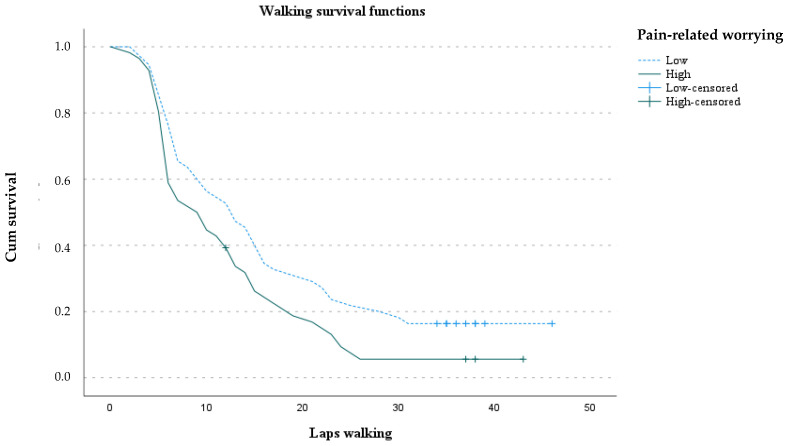
Walking maintenance along the walking task.

The mean number of laps was 14.3 (*SD* = 10.6) (range: 2–46) (Table 1). Analysis of the walking survival functions showed that the number of laps walked was higher for women with low scores on PCS than women with high scores (LongRank = 4.21; *p* = 0.04) (Figure 1).

However, pain-related worrying was not a significant predictor of walking persistence. As a result of univariate Cox regression analyses we explored goal preferences, pain-related worrying, age, fibromyalgia impact, and MET minutes/week. The estimated hazard ratios of the Cox regression analysis indicated that the risk of stopping walking increased as did the pain-avoidance goal preference score (*HR* = 1.29 [1.09–1.52], *p* = 0.002), controlling the fibromyalgia impact (*HR* = 1.02, [1.0–1.03], *p* = 0.014) (*χ^2^* = 14.85, *p* = 0.001).

#### 3.2.2. Total Walking Distance: Mediational Model of Pain-related Worrying through Goal Preference

**Figure 2:**
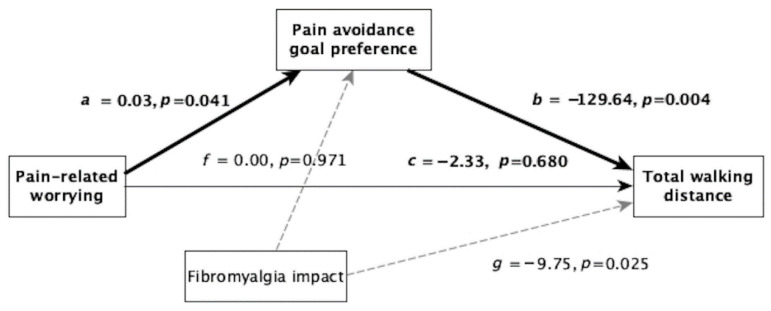
Paths linking pain-related worrying and total walking distance.

Given that fibromyalgia impact was a significant predictor of walking performance in terms of total distance covered, it was introduced in the analysis as a covariate. The total effect of the model was significant (*F* = 6.00, *p* = 0.003). The direct effect of the pain-related worrying total score on walking total distance was not significant (*c’* = −2.33, *p* = 0.680). However, we found a significant indirect effect through pain-avoidance goal preference (*ab* = −3.25 [−8.27–−0.060]) (Figure 2).

## 4. Discussion

Being physically active and doing exercise are well-documented practices for patients with fibromyalgia [6,62,63]. In fact, they are core treatment recommendations for this chronic pain problem and should be applied, tailored, graded, and progressed slowly while taking into account the patients’ exercise preferences and experiences [4,7]. Previous studies have shown that patients with fibromyalgia are motivated to engage in exercise [21,33] and start with this practice, but they usually do not follow through with it [21,22]. Therefore, as mentioned, physical activity and exercise maintenance appears to be a relevant clinical challenge. Consequently, in women with fibromyalgia who attended out-patient rehabilitation clinics, this study aimed to identify the encompassing role of pain-related worrying and goal preferences on walking persistence, taking into account the motivational reformulation of the fear avoidance model [25,26,27,28]. For this purpose we adapted the 6MWT to achieve a self-regulation frame and a maximum performance of 30 min, which is the minimum time recommended for walking exercise in our research [19,21,56].

As hypothesized, pain-related worrying introduced significant differences in goal preferences and walking persistence with the physical activity task. Women categorized as being high in pain-related worrying showed a stronger preference for pain avoidance goals relative to physical activity and exercise achievement goals, and worse performance in the physical activity task. However, the pain-related worrying did not play any role in the personal decision aimed at walking persistence. Although higher fibromyalgia impact, higher educational level, and the preference for pain-avoidance goals were significant predictors of stopping at the first bout, only goal preferences predicted the likelihood of stopping in the two following bouts. None of the included variables predicted the last decision to stop vs. to continue with the task, probably due to the few number of patients who reached this bout. The habitual preference for the pain-avoidance goal was also the main predictor of the task performance taking into account the number of laps. These results are in line with the tested mediational model, where pain-related worrying did not directly influence walking performance, but rather indirectly through the goal preferences, supporting a motivational explanation for walking maintenance in these patients. Moreover, it is in line with previous studies that have highlighted the association between pain catastrophizing and both the hedonic goal of avoiding pain or the assimilative way of coping which focuses the patients’ efforts on finding a solution to their pain problem [29,64,65]. Note that participants had a long-lasting pain and were diagnosed with fibromyalgia around six years ago. Despite this, taking into account their strong endorsement of pain-avoidance goals and the relationships of goal preferences with pain-related worrying, the patients appeared more perseverant in solving their insoluble chronic pain problems instead of focusing on different goals, such as exercising. In this vein, only a low proportion of patients (around 12%) completed the 30 min walking task. Fatigue and pain were the main reasons for abandoning the task and primarily pain in women with high pain-related worrying. Moreover, symptoms and illness impact were not associated with goal preferences but with pain-related worrying which, in turn, aims to avoid pain. Such results have implications on psychological interventions, as independently of the clinical situation of patients, it is possible to modify goal preferences targeting realistic and healthy aims such as physical activity and exercise.

Previous research using the original GPQ in people with different pain complaints has shown that endorsing either an achievement or pain-avoidance goals was associated with more disability [29]. In women with fibromyalgia, pain catastrophizing has shown a direct influence on self-reported disability and health impact, whereas the pain-avoidance goal preferences have shown an indirect influence through the activity avoidance pattern on the same variables [47]. Hence, the present study adds evidence about the role of these constructs in fibromyalgia showing, first of all, an indirect mechanism for pain-related worrying in the walking persistence and, secondly, the close link between goal preferences and behavior in these patients. Our findings have shown that pain-avoidance goal preferences influenced a more specific and objective way of avoidance behavior related to walking. Therefore, goal preferences appear to be a proximal determinant of the fibromyalgia patients’ physical activity behavior. Patients’ goal preference should be explored by health care providers when they prescribe physical activity and exercise, in order to adjust patients’ expectations about their symptoms when they undertake the activity and prevent both avoidance and escape behaviors.

The contribution of pain catastrophizing on disability and other health outcomes is well documented [34,37,38,58,66]. Moreover, in women with fibromyalgia, pain catastrophizing has been relevant in explaining the discordance between objective and subjective measures of physical function [18] and the patients’ physical fitness and active lifestyle [9]. However, to the best of our knowledge, there are few studies exploring specifically how catastrophizing influences physical activity and exercise or walking. Recently, Lazaridou and colleagues [41], using electronic diaries and a step count, found that increases in daily physical activity was associated with more pain intensity in women with fibromyalgia, mainly in those with a higher rate of catastrophizing. Hence, pain catastrophizing shows different ways of influence on physical activity and it is worth conducting further research about its role on walking exercise, mainly considering new recommendations about its assessment [31]. In women with fibromyalgia, from a motivational approach, the roles of catastrophizing and goal preferences have been explored in functional limitations. However, fatigue avoidance was one of the goal preferences relative to other achievement goals focused on several daily life tasks (work and homework) [67].

Taking into account descriptive data, women of this study reported high chronicity, pain intensity, fatigue, and fibromyalgia impact perception. Furthermore, participants scored high in pain-related worrying and showed a strong preference for short-term pain avoidance goals relative to physical activity and exercise long-term achievement goals. It is worth underlining that the decision to stop and performance in the walking task depended mainly on the patients’ goal preferences and not on chronicity and perceived symptoms, despite those symptoms being a reason to abandon the task. This finding is not in line with previous results where symptoms made significant contributions to physical fitness and physical activity [10,68,69]. Finally, fibromyalgia impact perception showed a significant relationship with walking performance in the same vein of previous research conducted with the 6MWT [51,70].

We should bear in mind some study limitations. First, the study has been conducted only with women who attended the tertiary level of health care, focused on rehabilitation treatment; hence, we should aim to replicate these findings in other patients with fibromyalgia. Second, the walking task was conducted inside the FU and is not fully representative of habitual walking activity or exercise. It is performed without natural stimuli that women may find outside; moreover, the task is performed in a repetitive manner, which could be considered dull and may potentially increase their likelihood of dropping out, although this was not a reported reason in this case. Finally, mediation analysis was cross-sectional and causation cannot be inferred. Furthermore, despite the statistical significance of some of its parameters, the total values were found to be low. Moreover, the number of statistical tests conducted could be considered excessive due to our sample size. Nevertheless, logistic and Cox regression and mediational model analyses are thought to explore different perspectives of the association of pain catastrophizing and goal preferences, and the results are coherent through them. Conversely, there are several strengths to highlight, such as the novel procedure of adapting a well-documented test (the 6MWT), the obtained objective measures, and the support for the main role of pain-related worrying in these women also in physical activity tasks. Our results show that its influence on walking persistence is mediated by the preference for short-term goals, such as pain avoidance, relative to physical activity and exercise long-term achievement goals, putting walking behavior in the motivational context of competing goals.

## 5. Conclusions

In fibromyalgia it is recommended to use individually tailored exercise programs [7] and, in this vein, health care professionals should take into account not only physical variables, but also motivational variables related to worries and goal preferences, because avoiding pain and maintaining exercise compete with each other in the context of physical activity tasks. Targeting physical activity and exercise implies working to reduce pain-related worrying and increases the value and commitment to physical activity and exercise goals.

## Figures and Tables

**Table 1:** Descriptive and difference analysis.

	Entire Sample				Low Pain-Related Worrying				High Pain-Related Worrying				
	*n* (%)	Mean	(SD)	[95% CI]	*n* (%)	Mean	(SD)	[95% CI]	*n* (%)	Mean	(SD)	[95% CI]	t
Pain-related worrying	111 (100)	30.9	(12.3)	[28.6–33.2]	55 (49.5)	20.7	(7.8)	[18.6–22.8]	56(50.5)	40.9	(6.2)	[39.2–42.5]	
Age (years)		51.4	(9.7)	[49.6–53.2]									ns
Time from symptoms		14.1	(9.8)	[12.1–16.0]									ns
Time from diagnosis		6.2	(6.2)	[5.0–7.4]									ns
Fatigue intensity b. t.^a^		7.3	(2.2)	[6.9–7.7]									ns
Pain intensity b. t.^a^		6.5	(2.2)	[6.1–6.9]		5.7	(2.1)	[5.2–6.3]		7.3	(2.1)	[6.8–7.9]	−4.16 ***
Pain intensity		6.8	(1.7)	[6.5–7.1]		6.1	(1.6)	[5.7–6.5]		7.4	(1.5)	[7.0–7.8]	−4.68 ***
Fibromyalgia impact		72.3	(15.8)	[69.3–75.2]		66.0	(17.2)	[61.4–70.2]		78.4	(11.6)	[75.3–81.5]	−4.44 ***
Goal preferences		4.4	(1.3)	[4.2–4.7]		4.1	(1.4)	[3.8–4.5]		4.7	(1.2)	[4.4–5.1]	−2.44 *
Total MET-min/week ^b^		694	(1635)	[439–873]		856	(1701)	[509–1506]		384	(879)	[231–693]	4.76 * c
Number of laps		14.3	(10.6)	[12.3–16.3]		16.4	(11.7)	[13.2–19.6]		12.3	(9.1)	[9.8–14.7]	2.07 *
Walking distance ^d^:													
Total		886	(637)	[766–1006]		1010	(702)	[820–1200]		765	(546)	[619–911]	2.05 *
Bout 1	111 (100)	396	(85)	[380–412]	55 (49.5)	420	(77)	[399–441]	56(50.5)	372	(86)	[349–395]	3.14 **
Bout 2	65 (58.6)	396	(91)	[374–419]	34 (61.8)	423	(80)	[396–452]	31(55.4)	366	(95)	[332–401]	2.65 **
Bout 3	35 (31.5)	412	(86)	[383–442]	19 (54.3)				16(45.7)				ns
Bout 4	20 (18.0)	425	(86)	[384–465]	13 (65.0)				7 (35.0)				ns
Bout 5	13 (11.7)	443	(107)	[379–508]	9 (69.2)				4 (30.8)				ns

^a^ = before the task; **^b^** = Median (Interquartile Rank); **^c^** = npar median test; **^d^** = number of meters rounded; * *p* < 0.05; ** *p* < 0.01; *** *p* < 0.001.

**Table 2:** Logistic regression analyses.

Stop vs. Walking						
Model ^a^	*n* ^b^	*OR*	[95% CI]	*p*	*χ^2^* ^c^	Cp
1st bout	111/46					
Goals preference		1.443	[1.026–2.030]	0.027	13.467 **	3.28
Fibromyalgia impact		1.029	[1.000–1.060]	0.037		
Educational level		2.428	[1.055–5.589]	0.033		
2nd bout	65/30					
Goals preference		1.621	[1.166–2.255]	0.003	11.295 **	4.04
Educational level		2.300	[0.957–5.530]	0.058		
3rd bout	35/10					
Goals preference		1.540	[1.073–2.210]	0.018	5.617 *	1.78

^a^: In table only variables in the final equations; ^b^: in each bout: n of participants/n who stopped after the bout; *OR* = odds ratio; ^c^: test of model coefficient; * *p* < 0.05 **; *p* < 0.01.

## Data Availability

Data are available by request to the corresponding author (slroig@umh.es).
